# HPV vaccination has not increased sexual activity or accelerated sexual debut in a college-aged cohort of men and women

**DOI:** 10.1186/s12889-019-7134-1

**Published:** 2019-06-25

**Authors:** Andrew F. Brouwer, Rachel L. Delinger, Marisa C. Eisenberg, Lora P. Campredon, Heather M. Walline, Thomas E. Carey, Rafael Meza

**Affiliations:** 1 Department of Epidemiology, University of Michigan, 1415 Washington Heights, Ann Arbor, MI 48109 USA; 2 Department of Otolaryngology, University of Michigan, 1500 E. Medical Center Dr., Ann Arbor, MI 48109 USA

**Keywords:** Human papillomavirus, Sexual behavior, HPV vaccine, College-aged population, Cohort study, M-HOC study

## Abstract

**Background:**

The human papillomavirus (HPV) is the most common sexually transmitted infection and is linked to several types of cancer. HPV vaccination uptake in the U.S. is relatively low, despite the vaccine’s high efficacy. Some parents of adolescents have concerns that vaccination will encourage sexual behavior and therefore choose not to vaccinate. Previous studies investigating vaccination and sexual behavior have included only young women and girls.

**Methods:**

The objective of this study is to assess associations between HPV-vaccination and sexual behavior in a college-age cohort of both men and women. We analyzed questionnaire data collected from the Michigan HPV and Oropharyngeal Cancer Study, a cohort study designed to investigate HPV infection and its association with sexual behavior (data collected 2015–17, Ann Arbor, MI). Here, we consider vaccination status, sexual behavior, and substance use among 241 college-aged men and women. Logistic, Poisson, and Cox regression were used to determine the relationship between probability of sexual debut, number of sexual partners, and HPV vaccination status at baseline as well as between age at sexual debut and vaccination status at debut.

**Results:**

HPV vaccination status was not significantly associated with an increased likelihood of sexual debut (odds ratio: 0.80 (95% CI: 0.41–1.58), decreased age of sexual debut (hazard ratio: 0.81 (95% CI: 0.65–1.00), nor an increased number of sexual partners (per year sexually active; incidence rate ratio: 1.27 (95% CI: 0.86–1.87)) in this cohort, after controlling for age, race, sex, and substance use. Instead, race or alcohol use were independent predictors of sexual behavior.

**Conclusions:**

Concerns about the influence of the HPV vaccine on sexual behavior are likely unfounded for both men and women. These results can aid in increasing vaccine acceptability, inform and strengthen physician recommendations, and ultimately reduce the burden of HPV and HPV-related cancers in the U.S.

**Electronic supplementary material:**

The online version of this article (10.1186/s12889-019-7134-1) contains supplementary material, which is available to authorized users.

## Background

The human papillomavirus (HPV) is the most common sexually transmitted infection, with approximately 14 million new infections, both genital and oral, occurring every year in the United States; the vast majority of sexually active men and women will harbor an HPV infection at some point in their lifetime [1]. HPV infection with certain viral strains is linked to virtually every case of cervical cancer, 90% of anal cancers, and 40% of other genital cancers [2]. Additionally, while the burden of high-risk oral HPV infection in the population is low (around 4%) [3], oropharyngeal squamous cell carcinomas have surpassed cervical cancers as the most common HPV-attributed cancers [2]—indeed, over 80% of oropharyngeal cancers at the University of Michigan Health System have been found to contain high-risk (oncogenic) HPV (defined in that study as genotypes 16, 18, 31, 33, 35, 39, 45, 51, 52, 56, 58, 59, 66, 68, and 73) [4].

There are currently three prophylactic HPV vaccines with FDA approval: Gardasil (HPV types 6, 11, 16, and 18; approved in 2006 for young women, 2009 for young men), Cervarix (types 16 and 18; approved in 2009 for young women), and Gardasil 9 (types 6, 11, 16, 18, 31, 33, 45, 52, 58; approved in 2014 for young women and men). These vaccines target viral genotypes that are associated with a high risk of cervical cancer (HPV 16, 18, 31, 33, 45, 52, and 58) and those associated with genital warts (HPV 6 and 11). In the U.S., routine vaccination is recommended for 11–12 year old adolescents of both sexes. A two-dose vaccine schedule, given 6–12 months apart, is recommended for individuals who initiate the series between 9 and 14 years of age. A three-dose vaccination series is recommended for individuals who initiate the series between 15 and 26 years of age, as well as for immunocompromised persons [5]. Insurance in the US typically covers HPV vaccination for people within the recommended age ranges. Prevalence of vaccine genotypes is much lower in vaccinated people [6, 7].

Despite the high efficacy and safety of the vaccines [8], there has been low coverage and vaccine initiation rates among adolescents in the U.S., although coverage has recently been increasing, particularly in young men. In 2017, only 68.6% of young women and 62.6% of young men had initiated the vaccine sequence in the U.S., while only 53.1% of young women and 44.3% of young men had received the full three recommended doses [9]. This coverage lags behind other adolescent vaccines [9] and falls considerably short of the goal set forth by the U.S. Department of Health and Human Services that 80% of adolescents be vaccinated against HPV. The CDC estimates that achieving this target uptake would prevent an additional 53,000 cases of cervical cancer [10].

Several factors have contributed to the low HPV vaccine uptake. General barriers to adolescent vaccine uptake include fewer visits to healthcare providers, incorrect beliefs about vaccines and vaccine safety, parental attitudes, and missed opportunities for vaccine administration [11–13]. Providers often fail to strongly endorse the HPV vaccination—particularly for patients who were younger, male, or from an ethnic minority [14, 15]. Various personal and political stances were also found to be barriers for strong provider recommendations. Additionally, because HPV is a sexually-transmitted infection, many parents have expressed concern that HPV vaccination may encourage children to become sexually active or promote promiscuous or riskier sexual behavior. Several studies assessing parental opinions of the HPV vaccine have found that up to 20% of parents believe the vaccination would lead to riskier sexual behavior in the future [16]. Parents who cite attitudinal barriers to vaccination are less accepting of the vaccine than parents who cite logistical barriers to vaccination their children [17]. These factors are a major obstacle in HPV vaccine uptake. Increasing acceptability and understanding among parents, as well as strengthening provider recommendations, will be necessary for expanding vaccine coverage and reducing disease burden [18].

Previous studies have found no evidence that HPV vaccination impacts sexual behavior, risk perception, or sexually transmitted infection incidence [19–26]. However, these studies have focused only on adolescent and young adult women, largely because cultural concerns about vaccination affecting sexual behavior have been muted for men [27], which is reflective of the sexual double-standard. Here, we measure the association between HPV vaccination and sexual behaviors in male and female college-aged students at the University of Michigan and college-aged participants living in Ann Arbor, Michigan and the surrounding communities. The study participants are part of the Michigan HPV and Oropharyngeal Cancer (M-HOC) study, which aims to understand the natural history of oral HPV infection by collecting longitudinal questionnaire and biological data on a cohort from the Ann Arbor, Michigan area.

## Methods

### Ethics, consent and permissions

Written and documented informed consent was obtained from all participants. Participant ID numbers were assigned to ensure participant confidentiality. The University of Michigan IRB approved consent documents and study protocol (HUM00090236).

### Study participants

Electronic questionnaires were administered in a private room to a convenience sample of college students and adult residents of Ann Arbor, Michigan and the immediate surrounding areas. Study participants were recruited at University of Michigan campus dormitories, through fliers, and through the UM Health Research website. Volunteers over the age of 18 who were willing to return every 3–4 months for 3 years for follow-up visits were invited to enroll. Participants with a history of head and neck cancer were not eligible. Additional information is available in our study protocol [28]. Enrollment has been completed. Data analysis here was restricted to the baseline questionnaire of college-aged (18–22) participants (*N* = 241) to investigate the impact of adolescent HPV vaccination on sexual behavior (data collected 2015–17, analyzed 2017–19). Recruitment in this age range targeted younger people to maximize retention over the planned 3-year study. Certain analyses are restricted to participants who had or did not have the HPV vaccine (*N* = 217; those who were unsure or did not answer were excluded).

### Questionnaire

A baseline questionnaire was administered to each participant at their initial visit. The questionnaire was designed to individually assess a variety of topics including demographics, vaccination, sexual health and behavior, and alcohol and drug use. Sexual behavior questions separately assessed current and past experiences of vaginal, oral, and anal sex.

### Exposure and outcomes

Three key outcomes were considered: occurrence of sexual debut (the first experience of vaginal, oral, or anal sex), number of sexual partners (lifetime number or per year sexually active), and age at sexual debut. Sexual behavior variables define sexual contact as vaginal intercourse, oral sex, or anal sex and define a sexual partner as an individual with whom one engages in one or more of these acts.

The main exposure variables in this analysis were vaccination status at study baseline (“Have you been vaccinated against human papillomavirus (HPV)?”) and at sexual debut as determined by reported age at vaccination (“What age did you get your first dose [of HPV vaccine]?”) and at sexual debut (“How old were you when you first had [vaginal/oral/anal] sex?”). Participants were considered vaccinated at sexual debut if their reported age at first dose of an HPV vaccine was less than or equal to their age of sexual debut. Anecdotally, participants contacting parents for vaccination status confirmation during the baseline interview was not unusual.

Vaccination at baseline was used as the exposure for the occurrence of sexual debut and number of sexual partner outcomes because we were interested in whether there was an association between them, irrespective of the timing of vaccination and sexual debut. However, when analyzing the impact of vaccination on the age of sexual debut, we recognize that participants may have been vaccinated after sexual debut but before their baseline visit. Hence, we use vaccination at the time of sexual debut as the exposure variable for this analysis.

### Statistical analysis

Descriptive analysis was performed to assess participant characteristics and behaviors from questionnaire data, including demographics, vaccination, sexual behavior, and substance-use. Differences in numbers and ages by vaccination status or gender were assessed by t-test or ANOVA, while differences in distribution of categorical or binary variables were assessed by chi-square test. *P*-values are omitted if test assumptions appear to be violated. Logistic regression was used to assess the relationship between baseline HPV vaccination status and sexual debut; results are reported as odds ratios (i.e., exponentiated model coefficients). Poisson regression, with an offset of (the log of) time since sexual debut, was used to determine the association between baseline HPV vaccination status and number of sexual partners (per year since sexual debut); results are presented as incidence ratios (i.e., exponentiated model coefficients). When age at sexual debut was the same as the current age, we assumed that the time since sexual debut was 1/3 years (consistent with uniform distribution of events). Cox proportional hazard models and the log-rank test were used to assess the relationship between HPV vaccination status at sexual debut and age at sexual debut. Results are presented as hazard ratios (i.e., exponentiated model coefficients). Models were adjusted for various demographic and behavioral characteristics, including age, sex, race, alcohol, and drug use at the time of the survey. Both multivariate analyses were done on the subset of participants who reported having or not having the HPV vaccination at baseline. All analyses were done in R v3.4. Statistical significance is reported at level *α* = 0.05; associations in the multivariate models with *p*-values less than α = 0.1 are noted as approaching significance to avoid overlooking associations that were not significant because of limited sample size.

## Results

### Population characteristics and differences by vaccination status and gender

At completion of study enrollment, 241 college-aged participants were enrolled into the study and completed the baseline questionnaire. Participant demographics, vaccination status, substance use, and sexual behaviors are shown in Table 1. The mean age of the college-aged cohort was 18.9 years. The majority of participants were female (70%) and white (54%). A majority of participants (63%) had previous sexual experience at baseline, of whom 77% had a history of vaginal sex, 96% had a history of oral sex, and 20% had a history of anal sex. The mean age of sexual debut was 16.6 years; however, this value is likely an underestimate of the (eventual average) age of sexual debut for this cohort because some participants may debut after the survey.

**Table 1:** Characteristics of the M-HOC college-age cohort and stratification by HPV vaccination status and gender

	Full Cohort		Vaccinated		Unvaccinated			Female		Male		
Variable	N	Value	N	Value	N	Value	*p*-value^a^	N	Value	N	Value	*p*-value^a^
Age	241	18.9 (1.0)	158	18.8 (1.0)	59	19.1 (1.0)	0.09	168	18.8 (1.0)	73	19.0 (1.1)	0.17
Female	241	70% (168)	158	74% (117)	59	66% (39)	0.25	168	100% (168)	73	0% (0)	–
Race	241		158		59		–	168		73		–
White		54% (131)		56% (88)		61% (36)			56% (94)		52% (37)	
Asian		31% (74)		30% (47)		24% (14)			29% (49)		35% (25)	
Black		6% (14)		4% (7)		8% (5)			5% (9)		7% (5)	
Other/multiracial		8% (20)		9% (14)		7% (4)			10% (16)		6% (4)	
Vaccinated	217	73% (158)	158	100% (153)	59	0% (0)	–	156	75% (117)	61	67% (41)	0.25
Age at first vaccine dose	–	–	102	15.5 (2.4)	–	–	–	79	15.0 (2.5)	22	16.7 (1.6)	**<0.001**
Current smoker	231	1% (2)	158	1% (1)	59	2% (1)	–	168	0% (0)	73	3% (2)	–
Alcohol: current user	231	65% (157)	158	68% (104)	59	63% (35)	0.49	168	65% (109)	73	66% (48)	0.90
Alcohol: ever binge drinker^b^	231	15% (37)	158	16% (25)	59	15% (9)	0.92	168	11% (19)	73	25% (18)	**0.01**
Marijuana: ever user	231	38% (91)	158	37% (58)	59	41% (24)	0.59	168	36% (60)	73	42% (31)	0.32
Has had any sex	231	63% (151)	158	64% (101)	59	61% (36)	0.69	168	62% (104)	73	64% (47)	0.71
If has had sex, has had												
Vaginal	151	77% (116)	101	77% (78)	36	75% (27)	0.79	104	78% (81)	47	74% (35)	0.70
Oral	151	96% (145)	101	97% (93)	36	94% (31)	–	104	97% (101)	47	94% (44)	–
Anal	151	20% (30)	101	21% (21)	36	22% (8)	0.86	104	17% (18)	47	26% (12)	0.24
Lifetime number of sexual partners												
Any	241	2.2 (3.7)	158	2.7 (4.2)	59	1.9 (2.7)	0.15	168	2.1 (3.2)	73	2.8 (4.6)	0.24
Vaginal sex	241	1.6 (3.1)	158	1.9 (3.5)	59	1.2 (2.3)	0.09	168	1.7 (2.9)	73	1.4 (3.6)	0.58
Oral sex	241	2.3 (3.6)	158	2.7 (4.1)	59	1.8 (2.7)	0.09	168	2.1 (3.0)	73	2.7 (4.7)	0.34
Anal sex	241	0.2 (0.8)	158	0.3 (0.8)	59	0.2 (0.6)	0.64	168	0.1 (0.5)	73	0.4 (1.2)	0.08
Age at sexual debut	150	16.6 (1.7)	101	16.5 (1.7)	36	16.9 (1.8)	0.18	104	16.7 (1.6)	46	16.5 (1.9)	0.58
Uses protection during vaginal sex	114		78		26		–	81		33		–
Always		47% (54)		41% (32)		62% (16)			46% (37)		52% (17)	
Most of the time		32% (37)		37% (29)		23% (6)			33% (27)		30% (10)	
Sometimes		9% (10)		8% (6)		8% (2)			11% (9)		2% (1)	
Rarely		6% (7)		9% (7)		0% (0)			5% (4)		9% (3)	
Never		5% (6)		5% (4)		8% (2)			5% (4)		6% (2)	
Uses protection during oral sex	139		96		31		–	96		43		–
Always		1% (1)		1% (1)		0% (0)			1% (1)		0% (0)	
Most of the time		0% (0)		0% (0)		0% (0)			0% (0)		0% (0)	
Sometimes		4% (5)		4% (4)		3% (1)			4% (4)		2% (1)	
Rarely		8% (11)		9% (10)		0% (0)			10% (10)		2% (1)	
Never		88% (122)		84% (81)		97% (30)			84% (81)		95% (41)	
Uses protection during anal sex	30		21		8		–	18		12		–
Always		33% (10)		29% (6)		50% (4)			28% (5)		42% (5)	
Most of the time		17% (5)		14% (3)		13% (1)			17% (3)		17% (2)	
Sometimes		7% (2)		10% (2)		0% (0)			11% (2)		0% (0)	
Rarely		3% (1)		5% (1)		0% (0)			0% (0)		8% (1)	
Never		40% (12)		43% (9)		38% (3)			44% (8)		33% (4)	

Values are % (number) out of N or age/number (sd). Only 217 of the 241 participants indicated vaccination status. Because participants could choose to not answer questions, sums of categorical entries may not equal the corresponding N. The M-HOC (Michigan HPV and Oropharyngeal Cancer) study was conducted in 2015–17 in Ann Arbor, MI

a: *p*-value for difference in means between vaccinated and unvaccinated populations, given by t-test (continuous or integer) or chi-square (binary and categorical). Boldface indicate *p* < 0.05

b: Habitual consumption of at least 6–9 drinks in a sitting

Most participants (90%) indicated their HPV vaccination status. Comparisons of participant characteristics and behaviors by vaccination status and gender are also shown in Table 1. Vaccinated individuals were more likely to be female, although this difference was not statistically significant. No significant differences in demographic characteristics were found between vaccination groups, nor in terms of substance use or use of protection during sex. The unadjusted difference in the mean number of vaginal sexual partners achieved statistical significance, with vaccinated individuals reporting a higher mean number of sexual partners, though it would be misleading to interpret this statistic without adjusting for covariates and time since sexual debut (see below). Men and women differed slightly (but not statistically significantly) in proportion vaccinated (67% vs. 75%), and men were vaccinated later on average (age 16.7 vs. 15.0). There were no significant differences in sexual behavior by gender (Table 1).

### Multivariate models

We analyzed sexual debut, the number of sexual partners, and age at sexual debut, controlling for age, sex, race, substance use, and vaccination status (Table 2). Analyses likelihood of sexual debut and number of sexual partners were restricted to participants who self-reported their HPV status (*N* = 217); analysis of age at sexual debut was restricted to participants who additionally reported age at vaccination and age at sexual debut (*N* = 161). Increased likelihood of sexual debut was associated with age and reporting current alcohol use; being white approached significance. (Note: although the positive association between being white and reporting current alcohol use approached significance (*p* = 0.07), the regression accounts for the correlation structure. Hence, these covariates are independent predictors of sexual debut). Vaccination status was not significantly associated with probability of sexual debut. Next, by using a Poisson regression with an offset of time since sexual debut, we were able to adjust our analysis of number of sexual partners to account for the most important factor, namely, the length of time participants had been sexually active. After this adjustment, the number of sexual partners was strongly positively associated with current alcohol use and weakly (in magnitude), negatively associated with being white. Vaccination status also approached statistical significance in this analysis; however, unlike in Table 1, it was weakly associated with fewer sexual partners after adjusting for covariates.

**Table 2:** Multivariate analyses predicting number of sexual partners and age at sexual debut

	Probability of sexual debut			Number of sexual partners (per year)			Age at sexual debut		
Covariate	Odds ratio	95%CI	*p*-value	Incidence ratio	95% CI	*p*-value	Hazard ratio	95% CI	*p*-value
Intercept	0.61	(0.19, 1.96)	0.41	1.47	(0.98, 2.19)	0.06	–	–	–
Age (per year over 18)	1.48	(1.05, 2.14)	**0.03**	0.97	(0.89, 1.05)	0.42	0.88	(0.73, 1.07)	0.21
Female	0.85	(0.42, 1.68)	0.65	0.99	(0.82, 1.20)	0.94	0.90	(0.58, 1.42)	0.65
Race: white	1.78	(0.98, 3.27)	0.06	0.75	(0.63, 0.89)	**0.001**	1.51	(1.01, 2.28)	**0.04**
Alcohol: current drinker	2.73	(1.36, 5.56)	**0.001**	1.81	(1.36, 2.43)	**<0.001**	1.65	(0.98, 2.80)	0.06
Alcohol: binge drinker	1.71	(0.64, 5.12)	0.30	0.99	(0.79, 1.22)	0.92	1.34	(0.82, 2.22)	0.25
Marijuana: ever user	1.27	(0.60, 2.70)	0.53	1.06	(0.86, 1.32)	0.57	1.13	(0.73,1.75)	0.58
Vaccinated									
At baseline	0.80	(0.41, 1.58)	0.52	0.81	(0.65, 1.00)	0.06	–	–	–
At sexual debut	–	–	–	–	–	–	1.27	(0.86, 1.87)	0.23

The probability of sexual debut is analyzed by logistic regression; values are given as odds ratios (exponentiated model coefficients). The number of sexual partners is analyzed with Poisson regression with an offset of (the log of) number of years sexually active; values are incidence rate ratios (exponentiated model coefficients). The age at sexual debut is analyzed by a Cox proportional hazard model; results given as hazard ratios (exponentiated model coefficients). Boldface indicate *p* < 0.05. Study conducted in 2015–17, Ann Arbor, MI

**Fig. 1:**
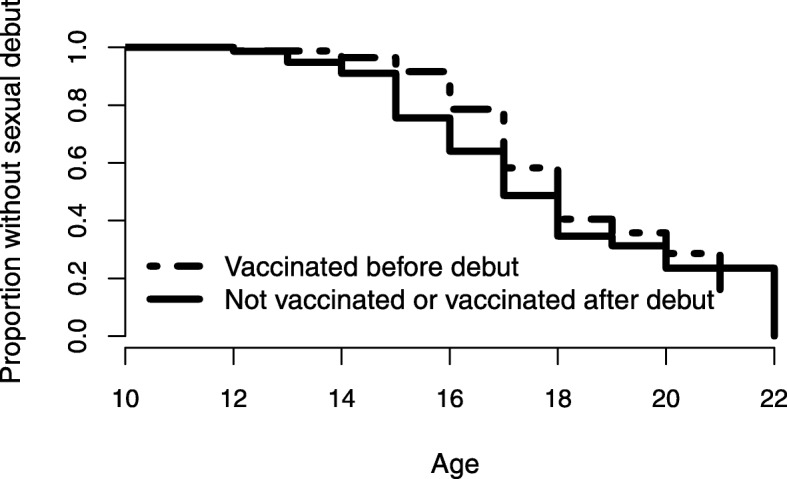
Kaplan-Meier plot of age at sexual debut stratified by HPV vaccination status at debut. No statistically significant difference between the curves was detected (log-rank test, *p* = 0.30)

For age of sexual debut, values are multiplicative effects on the underlying hazard rate, meaning that larger hazard ratios are associated with earlier age of sexual debut (Table 2). This analysis was restricted to the subset of participants who reported their age at vaccination or not being vaccinated; among this subset, 83 participants reported vaccination before or the same age as debut, and 78 participants reported vaccination after debut or no vaccination. There were 110 sexual debuts in this subset. Being white was associated with an earlier sexual debut, and reporting current alcohol use at baseline neared significance. But, vaccination status at sexual debut was not significant. A Kaplan-Meier plot of sexual debut stratified by HPV-vaccination status at sexual debut (Fig. 1) illustrates the finding that HPV-vaccination status at debut is not significantly associated with age of sexual debut (log-rank test, *p* = 0.30). Analogous multivariate models of probability of sexual debut, number of sexual partners and age of sexual debut for vaginal, oral, and anal sex are included in the Additional file 1. These models have largely similar interpretations, although the probability and age of vaginal sexual debut were more associated with current marijuana use than with current alcohol use. Binge drinking was not associated with any of the outcomes after accounting for current alcohol use.

## Discussion

Our analysis of behavioral survey data for a college-age cohort reveals a nuanced story of sexual behavior and HPV vaccination. Although vaccinated participants had more sexual partners overall, after adjusting for time since sexual debut and other covariates, we found instead that a weak association between HPV-vaccination and fewer sexual partners approached statistical significance. Although we expect that this association may be spurious, it may also be that vaccination uptake and fewer sexual partners are both associated with risk avoidance behavior. Regardless, we interpret this finding as evidence that HPV-vaccination is not associated with increased sexual activity. Vaccination status at sexual debut was not significantly associated with the probability of sexual debut or the age at sexual debut, though alcohol use or being white were. Gender was not significantly associated with any of the main outcomes but was significant in certain sex-act-specific outcomes.

This study contributes to the emerging evidence that HPV vaccination does not promote sexual behavior in young adults. Previous studies have reported no differences in the number of sexual partners [19, 25], age of sexual debut [19], sexual activity and condom use [20], risk perceptions [22, 26], STI and pregnancy incidence or testing [21, 23, 24] in girls and young women. Many parents have hesitated to vaccinate their children against HPV out of fear of risky or adverse sexual behavior outcomes later in life [16]. For example, a 2009 study by Kahn et al. [29] found that only 48% of mothers intended to vaccinate daughters under the age of 13, the target age group for HPV vaccination. Therefore, evidence refuting these concerns has clinical implications, since parental concern has found to be an important barrier to physician recommendation of vaccination [30]. Physician biases and concerns related to teen sexual behavior have also been reported, particularly as a barrier to male vaccination [17, 31]. Since physician recommendations, as well as parental acceptance of the vaccine, are vital to vaccine uptake [18], strengthening the evidence for the lack of association between HPV vaccination and subsequent sexual behavior is imperative.

Our finding that sexual behavior (number of partners and sexual debut) is strongly associated with alcohol use is consistent with many previous studies citing a link between drinking and increased sexual activity. For example, the Harvard College Alcohol Study (CAS) provided national data showing that college students who engaged in binge drinking were 2–3 times more likely to engage in unplanned sex and unprotected sex [32]. In this study, we found that binge drinking was not associated with the outcomes after adjusting for any alcohol consumption, although the definition of binge drinking varied slightly between studies. Moreover, Metrik et al. [33] found both alcohol use and marijuana use were independently associated with a greater likelihood of sexual encounters; we found that alcohol, though not marijuana, use was associated with our sexual behavior outcomes in our cohort.

This study provided a first look at the cohort data generated by the M-HOC study, which, at its completion, has the potential to inform many other aspects of HPV infection, including the relationship between longitudinal sexual behaviors, including debut, and oral HPV infection incidence, prevalence, and clearance. This study is the first to investigate the impact of HPV vaccination on sexual behavior that we are aware of that has included men. Further, targeting a college-aged sample can investigate any effects that vaccination may have closer to the age of sexual debut. Studying both short-term and long-term effects of vaccination status is critical to understanding the interplay between sexual behavior and vaccination and ultimately increasing vaccine uptake.

Our analysis is based on self-reported questionnaire data, and it is not possible to independently confirm the reported sexual and substance-use behaviors or vaccination status. Since the majority of vaccinated participants in our cohort were vaccinated years prior to the baseline questionnaire, it is possible that individuals misremembered or were unsure of their HPV vaccination status. Although we accounted for HPV status at sexual debut by including it as an exposure, sexual debut and vaccination can both be viewed as survival processes, and thus future work could take a competing risks approach to assessing associations between them. Moreover, in this retrospective analysis, we tested for associations between substance use at study baseline and prior sexual debut; consequently, these associations are inherently not causative. Too, the location and recruiting method of this study may lead to a generalizability limitation. This cohort is composed of college-aged individuals, the majority of whom are white and attend a large university. These characteristics could potentially influence behavioral decisions in a manner not generalizable to other individuals of the same age. Moreover, because of the timing of vaccine introduction, this cohort was vaccinated later (mean age 15.5) than younger cohorts will likely be, since they did not (and largely could not) have received the vaccine as now recommended. Nonetheless, our results provide additional evidence of the lack of association between HPV vaccination and sexual behaviors across different populations.

## Conclusion

Our findings contribute to a fast-growing body of literature that demonstrates that HPV vaccination does not impact sexual behavior of adolescents or college-aged individuals. These studies are an important step to increasing vaccine uptake in the United States. By educating parents of adolescents as well as increasing awareness among physicians, physician recommendations can more accurately reflect the true benefits and risks of HPV vaccination, potentially leading to increased vaccine acceptability. We will continue follow-up of this cohort to further investigate behavioral outcomes and the natural history of HPV.

## Additional file

Additional file 1: **Table S1.** Characteristics of the M-HOC college-age cohort stratified by the number of sexual partners. **Table S2.** Characteristics of the M-HOC college-age cohort stratified by sexual experience. **Table S3.** Multivariate analyses predicting age at vaginal sexual debut and lifetime number of vaginal sexual partners. **Table S4.** Multivariate analyses predicting age at oral sexual debut and lifetime number of oral sexual partners. **Table S5.** Multivariate analyses predicting age at anal sexual debut and lifetime number of anal sexual partners. (DOCX 49 kb)

## Data Availability

The datasets generated and/or analyzed during the current study are not publicly available because of participant privacy concerns but are available from the corresponding author on reasonable request (IRB approval or a data use agreement may be required).

## Abbreviations

ANOVA: analysis of variance
CDC: Centers for Disease Control and Prevention
FDA: Food and Drug Administration
HPV: human papillomavirus
M-HOC: Michigan HPV and Oropharyngeal Cancer)
UM UM: University of Michigan
