# Supplementary material for: HPV vaccination has not increased sexual activity or accelerated sexual debut in a college-aged cohort of men and women
Source: BMC Public Health. 2019 Jun 25;19:821. doi: 10.1186/s12889-019-7134-1 (PMC6593582; doi:10.1186/s12889-019-7134-1)
Supplement: Supplementary file 1 — Table S1. Characteristics of the M-HOC college-age cohort stratified by the number of sexual partners. Table S2. Characteristics of the M-HOC college-age cohort stratified by sexual experience. Table S3. Multivariate analyses predicting age at vaginal sexual debut and lifetime number of vaginal sexual partners. Table S4. Multivariate analyses predicting age at oral sexual debut and lifetime number of oral sexual partners. Table S5. Multivariate analyses predicting age at anal sexual debut and lifetime number of anal sexual partners. (DOCX 49 kb) [file 12889_2019_7134_MOESM1_ESM.docx]

# Supplement

In this supplement, we characterize the populations by number of life-time sexual partners and by sexual experience. Then, we provide multivariate analyses of site-specific (vaginal, oral, anal) lifetime number of sexual partners and age at site-specific sexual debut.

Table S1: **Characteristics of the M-HOC college-age cohort stratified by the number of sexual partners.** Values are % (N) or age/number (sd).

|  | Lifetime number of sexual partners | | | | p-value* |
| --- | --- | --- | --- | --- | --- |
|  | 0 (N= 89) | 1 (N=65) | 2–5 (N=53) | 6+ (N=34) |  |
| Age | 18.6 (0.7) | 19.0 (1.1) | 18.9 (1.1) | 19.5 (1.2) | **<0.001** |
| Female | 72% (64) | 65% (42) | 77% (41) | 62% (21) | 0.33 |
| Race |  |  |  |  | **—** |
| White | 44% (39) | 62% (40) | 62% (33) | 56% (19) |  |
| Asian | 45% (40) | 26% (17) | 21% (11) | 18% (6) |  |
| Black | 4% (4) | 6% (4) | 6% (3) | 9% (3) |  |
| Other/multiracial | 6% (5) | 6% (4) | 11% (6) | 18% (6) |  |
| Vaccinated** | 72% (57) | 70% (39) | 73% (35) | 79% (27) | 0.79 |
| Age at first vaccine dose | 15.2 (2.5) | 16.3 (2.2) | 15.0 (2.3) | 14.7 (2.8) | 0.10 |
| Alcohol: current user | 44% (39) | 69% (45) | 79% (42) | 91% (31) | *<***0.001** |
| Alcohol: ever binge drinker | 9% (8) | 15% (10) | 17% (9) | 29% (10) | **0.04** |
| Marijuana: ever user | 23% (20) | 32% (21) | 55% (29) | 62% (21) | *<***0.001** |
| Age at sexual debut | — | 17.2 (1.5) | 16.6 (1.4) | 15.5 (1.9) | **<0.001** |
| *: p-value for difference among groups (ANOVA) or chi-square (categorical data).  **: Underling Ns are 79, 56, 48, and 34, respectively. | | | | | |

Table S2: **Characteristics of the M-HOC college-age cohort stratified by sexual experience.** Values are % (N) or age/number (sd).

|  | Vaginal sex (N=116) | p-value* | Oral sex (N=145) | p-value* | Anal sex (N=30) | p-value* |
| --- | --- | --- | --- | --- | --- | --- |
| Age | 19.0 (1.1) | **<0.001** | 19.1 (1.1) | **<0.001** | 19.3 (1.4) | **0.01** |
| Female | 70% (81) | 0.75 | 70% (101) | 0.71 | 60% (18) | 0.25 |
| Race: |  | **0.002** |  | **0.002** |  | — |
| White | 57% (66) |  | 60% (87) |  | 53% (16) |  |
| Asian | 22% (25) |  | 22% (32) |  | 23% (7) |  |
| Black | 9% (10) |  | 7% (10) |  | 7% (2) |  |
| Other/multiracial | 13% (15) |  | 11% (16) |  | 17% (5) |  |
| Vaccinated** | 74% (78) | 0.75 | 74% (98) | 0.74 | 72% (21) | 0.98 |
| Age at first vaccine dose | 15.2 (2.5) | 0.95 | 15.5 (2.4) | 0.70 | 15.4 (2.7) | 0.88 |
| Alcohol: current user | 78% (91) | *<***0.001** | 78% (113) | *<***0.001** | 87% (26) | *<***0.001** |
| Alcohol: binge drinker | 21% (24) | **0.02** | 19% (28) | **0.02** | 17% (5) | 0.32 |
| Marijuana: ever user | 54% (63) | *<***0.001** | 46% (66) | *<***0.001** | 57% (17) | **0.002** |
| Age at sexual debut | 16.6 (1.5) | — | 16.6 (1.7) | — | 16.0 (2.3) | — |
| Lifetime number of sexual partners*** | 3.9 (4.1) | — | 3.8 (4.1) | — | 7.5 (6.4) | — |
| *: p-value for comparison to those with no sexual experience (Table 2). | | | | | | |
| **: Underling Ns are 105, 132, and 29, respectively. | | | | | | |
| ***: Vaginal, oral, or anal sex partners. | | | | | | |

Table S3: **Multivariate analyses predicting age at vaginal sexual debut and lifetime number of vaginal sexual partners.** The probability of sexual debut is analyzed by logistic regression; values are given as odds ratios (exponentiated model coefficients). The number of sexual partners is analyzed with Poisson regression with an offset of (the log of) number of years sexually active; values are incidence rate ratios (exponentiated model coefficients). The age at sexual debut is analyzed by a Cox proportional hazard model; results given as hazard ratios (exponentiated model coefficients).

|  | Probability of sexual debut | | | Number of sexual partners (per year) | | | Age at sexual debut | | |
| --- | --- | --- | --- | --- | --- | --- | --- | --- | --- |
| Covariate | Odds ratio | 95%CI | p-value | Incidence ratio | 95% CI | p-value | Hazard ratio | 95% CI | p-value |
| Intercept | 0.41 | (0.13, 1.28) | 0.12 | 1.29 | (0.79, 2.08) | 0.31 | — | — | — |
| Age (per year over 18) | 1.33 | (0.98, 1.82) | 0.07 | 0.90 | (0.82, 0.99) | **0.04** | 0.97 | (0.78, 1.21) | 0.78 |
| Female | 1.12 | (0.67, 2.18) | 0.73 | 1.38 | (1.09, 1.77) | **0.008** | 1.30 | (0.77, 2.19) | 0.32 |
| Race: white | 1.21 | (0.62, 2.18) | 0.52 | 0.66 | (0.53, 0.82) | *<***0.001** | 1.29 | (0.81, 2.04) | 0.28 |
| Alcohol: current drinker | 1.52 | (1.36, 2.49) | 0.24 | 2.48 | (1.75, 3.55) | *<***0.001** | 1.11 | (0.62, 2.01) | 0.72 |
| Alcohol: binge drinker | 1.34 | (0.56, 3.31) | 0.51 | 1.15 | (0.90, 1.48) | 0.25 | 1.32 | (0.74, 2.35) | 0.35 |
| Marijuana: ever user | 2.87 | (1.45, 5.79) | **0.003** | 0.92 | (0.72, 1.20) | 0.54 | 1.72 | (1.04, 2.82) | **0.03** |
| Vaccinated |  |  |  |  |  |  |  |  |  |
| At baseline | 0.76 | (0.61, 1.47) | 0.42 | 0.77 | (0.58, 1.00) | 0.06 | — | — | — |
| At sexual debut | — | — | — | — | — | — | 1.28 | (0.83, 1.97) | 0.27 |

Table S4: **Multivariate analyses predicting age at oral sexual debut and lifetime number of oral sexual partners.** The probability of sexual debut is analyzed by logistic regression; values are given as odds ratios (exponentiated model coefficients). The number of sexual partners is analyzed with Poisson regression with an offset of (the log of) number of years sexually active; values are incidence rate ratios (exponentiated model coefficients). The age at sexual debut is analyzed by a Cox proportional hazard model; results given as hazard ratios (exponentiated model coefficients).

|  | Probability of sexual debut | | | Number of sexual partners (per year) | | | Age at sexual debut | | |
| --- | --- | --- | --- | --- | --- | --- | --- | --- | --- |
| Covariate | Odds ratio | 95%CI | p-value | Incidence ratio | 95% CI | p-value | Hazard ratio | 95% CI | p-value |
| Intercept | 0.54 | (0.17, 1.72) | 0.31 | 1.56 | (1.04, 2.32) | **0.03** | — | — |  |
| Age (per year over 18) | 1.46 | (1.05, 2.09) | **0.03** | 1.01 | (0.93, 1.10) | 0.71 | 0.90 | (0.75, 1.10) | 0.31 |
| Female | 1.01 | (0.52, 1.96) | 0.97 | 0.94 | (0.78, 1.14) | 0.56 | 0.94 | (0.59, 1.49) | 0.79 |
| Race: white | 1.58 | (0.87, 2.87) | 0.13 | 0.80 | (0.67, 0.96) | **0.01** | 1.40 | (0.92, 2.11) | 0.11 |
| Alcohol: current drinker | 2.97 | (1.49, 6.05) | **0.002** | 1.71 | (1.30, 2.28) | *<***0.001** | 1.83 | (1.06, 3.16) | **0.03** |
| Alcohol: binge drinker | 1.63 | (0.64, 4.59) | 0.32 | 0.87 | (0.69, 1.08) | 0.22 | 1.38 | (0.84, 2.29) | 0.21 |
| Marijuana: ever user | 1.08 | (0.52, 2.25) | 0.83 | 0.96 | (0.78, 1.18) | 0.72 | 1.07 | (0.69, 1.67) | 0.75 |
| Vaccinated |  |  |  |  |  |  | — | — | — |
| At baseline | 0.77 | (0.39, 1.50) | 0.43 | 0.84 | (0.67, 1.03) | 0.12 |  |  |  |
| At sexual debut | — | — | — | — | — | — | 1.18 | (0.80, 1.76) | 0.40 |

Table S5: **Multivariate analyses predicting age at anal sexual debut and lifetime number of anal sexual partners.** The probability of sexual debut is analyzed by logistic regression; values are given as odds ratios (exponentiated model coefficients). The number of sexual partners is analyzed with Poisson regression with an offset of (the log of) number of years sexually active; values are incidence rate ratios (exponentiated model coefficients). The age at sexual debut is analyzed by a Cox proportional hazard model; results given as hazard ratios (exponentiated model coefficients).

|  | Probability of sexual debut | | | Number of sexual partners (per year) | | | Age at sexual debut | | |
| --- | --- | --- | --- | --- | --- | --- | --- | --- | --- |
| Covariate | Odds ratio | 95%CI | p-value | Incidence ratio | 95% CI | p-value | Hazard ratio | 95% CI | p-value |
| Intercept | 0.08 | (0.01, 0.45) | **0.004** | 1.74 | (0.32, 8.25) | 0.50 | — | — |  |
| Age (per year over 18) | 1.47 | (1.02, 2.12) | **0.04** | 0.92 | (0.71, 1.17) | 0.52 | 0.90 | (0.59, 1.39) | 0.66 |
| Female | 0.48 | (0.20, 1.16) | 0.10 | 0.58 | (0.28, 1.24) | 0.16 | 0.38 | (0.20, 1.13) | 0.09 |
| Race: white | 0.83 | (0.36, 1.94) | 0.67 | 1.07 | (0.53, 2.13) | 0.85 | 0.64 | (0.28, 1.49) | 0.30 |
| Alcohol: current drinker | 3.26 | (1.02, 12.6) | 0.06 | 0.92 | (0.26, 3.84) | 0.90 | 4.20 | (1.09, 16.2) | **0.04** |
| Alcohol: binge drinker | 0.54 | (0.15, 1.61) | 0.29 | 0.56 | (0.22, 1.41) | 0.23 | 0.55 | (0.18, 1.74) | 0.31 |
| Marijuana: ever user | 1.30 | (0.50, 3.38) | 0.59 | 1.22 | (0.52, 2.80) | 0.64 | 0.72 | (0.36, 2.33) | 0.86 |
| Vaccinated |  |  |  |  |  |  |  |  |  |
| At baseline | 0.90 | (0.33, 2.22) | 0.82 | 1.00 | (0.44, 2.16) | 0.99 | — | — | — |
| At sexual debut | — | — | — | — | — | — | 1.16 | (0.50, 2.66) | 0.73 |
